# Defining Weight‐Loss‐Independent Hepatic Effects of Incretin‐Based Therapies

**DOI:** 10.1111/dom.71391

**Published:** 2026-09-24

**Authors:** Mingyuan Zhang, Hanyang Liu, Yawen Hao, Lanlan Chen

**Affiliations:** ^1^ Department of Hepatology and Gastroenterology Charité—Universitätsmedizin Berlin, Campus Virchow‐Klinikum (CVK) and Campus Charité Mitte (CCM) Berlin Germany; ^2^ Department of Hepatology, Jilin Provincial Key Laboratory of Metabolic Liver Diseases The First Hospital of Jilin University, Jilin University Changchun China; ^3^ China‐Singapore Belt and Road Joint Laboratory on Liver Disease Research The First Hospital of Jilin University, Jilin University Changchun China; ^4^ Department of Hepatobiliary and Pancreatic Surgery, General Surgery Center The First Hospital of Jilin University Changchun China

**Keywords:** antidiabetic drug, antiobesity drug, fatty liver disease, incretin therapy, weight management

Incretin‐based therapies are rapidly reshaping treatment for metabolic dysfunction‐associated steatotic liver disease (MASLD) and metabolic dysfunction‐associated steatohepatitis (MASH) [1]. Semaglutide has demonstrated histological efficacy in MASH and been approved for MASH with moderate‐to‐advanced fibrosis [2], whereas glucagon‐like peptide‐1 receptor (GLP‐1R)/glucagon receptor (GCGR) co‐agonists (e.g., survodutide and pemvidutide) are expanding the therapeutic landscape [3, 4]. As these agents produce both hepatic improvement and weight loss, a central mechanistic question has become increasingly prominent: How much of the liver benefit is mediated by weight reduction, and how much reflects pathways that are independent of weight loss?

This distinction matters for more than mechanism. If hepatic efficacy is partly independent of weight reduction, benefits might extend to people who lose relatively little weight, inform dose selection and support development of organ‐targeted incretin therapies. Conversely, over‐attributing hepatic responses to direct drug action could mislead trial interpretation and drug development. We therefore argue that ‘weight‐loss independence’ should not be treated as a binary property of a drug. Rather, it is an endpoint‐specific causal estimand that requires explicit separation of total, weight‐mediated and remaining treatment effects (Figure 1A).

**FIGURE 1:**
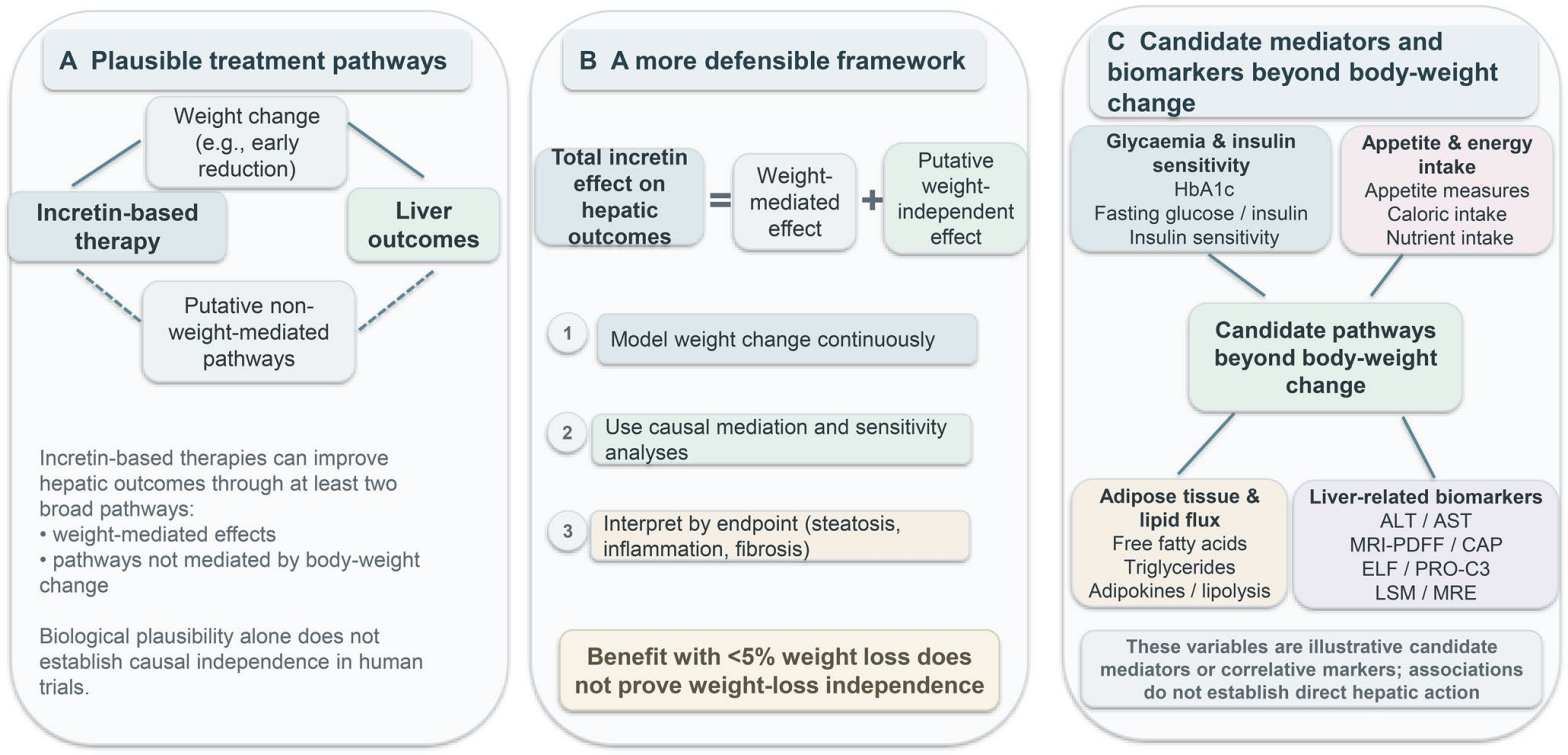
Causal framework for evaluating weight‐loss‐independent hepatic effects of incretin‐based therapies. (A) Incretin‐based therapies may improve liver outcomes through weight‐mediated and putative non‐weight‐mediated pathways; post‐randomization subgroup findings can be clinically descriptive but are not causal proof. (B) A more rigorous approach separates the total treatment effect into weight‐mediated and putative weight‐independent components using continuous weight modelling, causal mediation and endpoint‐specific analyses. (C) Candidate mediators and correlative biomarkers beyond body‐weight change include measures of glycaemia and insulin sensitivity, appetite and energy intake, adipose‐tissue lipid flux, and liver‐related biomarkers. These variables are illustrative; associations do not by themselves establish direct hepatic action. Abbreviations: ALT, alanine aminotransferase; AST, aspartate aminotransferase; CAP, controlled attenuation parameter; ELF, enhanced liver fibrosis test; HbA1c, glycated haemoglobin; LSM, liver stiffness measurement; MRE, magnetic resonance elastography; MRI‐PDFF, magnetic resonance imaging–proton density fat fraction; PRO‐C3, N‐terminal propeptide of type III collagen.

## 1 Post‐Randomization Subgrouping Does Not Establish Weight‐Loss Independence

The recent Phase 2 DD01 trial, a novel GLP‐1R/GCGR co‐agonist, illustrates the challenge. Its prespecified primary endpoint was the proportion of participants achieving at least 30% relative reduction in liver fat by MRI proton density fat fraction (MRI‐PDFF) at Week 12; body weight was an exploratory outcome and the < 5% weight‐loss subgroup analysis was post hoc [5]. After 12 weeks, mean body weight decreased by 3.8% with DD01 versus 0.4% with placebo and 14 (42%) of 33 DD01‐treated participants achieved at least 5% weight loss [5]. Hepatic benefits among participants losing < 5% of body weight are clinically encouraging and hypothesis‐generating, but this subgroup should not be interpreted as establishing causal independence from weight loss. Achieved weight change is a post‐randomization variable affected by treatment. Restricting an analysis to participants below an arbitrary weight‐loss threshold no longer preserves the original randomized comparison. Participants who remain below 5% weight loss after active treatment might differ from placebo participants meeting the same threshold in adherence, treatment exposure, baseline metabolic phenotype, appetite response, disease severity or other determinants of hepatic outcomes. Conditioning on a treatment‐affected variable can therefore introduce selection or collider bias, in which restricting on a variable influenced by both treatment and prognostic factors creates spurious associations between them [6]. Dichotomization also discards information: Participants losing 0.5% and 4.9% of body weight are assigned to the same category despite markedly different metabolic responses.

The causal question should instead be defined explicitly. The total treatment effect asks whether randomization to the drug improves a hepatic endpoint. A mediation question asks how much of this effect operates through weight change. A direct‐effect question asks what treatment effect would remain under a defined intervention on the mediator. These questions are related but not interchangeable. For each endpoint, analyses should report effect estimates on a prespecified scale, together with confidence intervals and appropriate statistical tests, rather than relying on qualitative labels. Importantly, a residual treatment effect after accounting for weight loss should initially be described as a putative weight‐independent effect, rather than automatically interpreted as evidence of direct hepatic receptor signalling (Figure 1B).

## 2 Weight Mediation Is Endpoint Specific

The recent mediation analysis of survodutide provides an important counterpoint. Among 170 participants with F2‐F3 MASH and paired biopsies, the estimated proportion of the treatment effect mediated by weight reduction was 58.2% for MRI‐PDFF, 77.4% for controlled attenuation parameter, but only 36.3% for fibrosis improvement [7]. These values were reported as point estimates, without confidence intervals for the mediation proportions themselves, and should therefore be interpreted cautiously. The qualitative pattern—greater mediation for steatosis‐related endpoints and lower mediation for fibrosis improvement—is more informative than the exact percentages.

These findings illustrate why a single label (‘weight dependent’ or ‘weight independent’) is unlikely to adequately characterize an entire drug. Different liver endpoints reflect different biological processes and operate on different timescales. Liver fat can change rapidly with energy balance and substrate flux, whereas inflammation and fibrosis reflect immune, vascular, stellate‐cell and extracellular‐matrix processes that might respond differently [8]. A therapy could therefore be largely weight mediated for steatosis but only partly weight mediated for fibrosis. Weight‐loss independence should consequently be defined for individual endpoints (e.g., MRI‐PDFF, MASH resolution or fibrosis improvement) rather than assigned globally to a therapeutic agent.

Biological evidence can support mechanistic plausibility but should not be conflated with causal mediation in humans. In mouse models resistant to GLP‐1R‐mediated weight loss, semaglutide still improved MASLD, whereas loss of GLP‐1Rs in liver sinusoidal endothelial cells substantially disrupted its hepatoprotective effects despite preserved weight reduction [9]. These findings support the biological plausibility of intrahepatic GLP‐1R‐dependent pathways in experimental models, although their relevance to weight‐independent hepatic effects in humans remains uncertain. Importantly, ‘not mediated by weight loss’ is not synonymous with ‘directly acting on the liver’. A putative weight‐independent component in humans could also operate through systemic or extrahepatic processes, including changes in glycaemia, insulin sensitivity, adipose‐tissue lipolysis, nutrient intake, glucagon signalling, systemic inflammation or inter‐organ communication [10] (Figure 1C). Thus, evidence for incomplete weight mediation should be viewed as hypothesis‐generating rather than as proof of a specific intrahepatic pathway.

## 3 A Framework for Future MASLD Trials

Future MASLD trials should prospectively define the causal question and collect the data required to answer it. First, weight change should be analysed continuously and, whenever possible, longitudinally rather than reduced to a threshold. Repeated measurements can help establish the temporal sequence between treatment exposure, weight change and hepatic responses. However, when weight change and hepatic improvement are measured at the same time point, it may be difficult to determine whether weight change is a true mediator or whether both reflect common upstream processes such as reduced caloric intake or improved insulin sensitivity. Baseline values of potential mediators and major prognostic variables should also be collected because randomization protects estimation of the treatment‐outcome relationship but does not randomize the mediator.

Second, causal mediation analyses should estimate the total, indirect (weight‐mediated) and remaining direct effects on a prespecified effect scale, together with effect estimates, 95% confidence intervals and appropriate statistical tests. The proportion mediated may be used to summarize the relative contribution of the indirect pathway, but its uncertainty should also be quantified using appropriate confidence intervals. Prespecified significance thresholds and sensitivity analyses should accompany these estimates. Such analyses depend on assumptions about mediator‐outcome confounding, which is particularly relevant to incretin therapies, because treatment simultaneously alters food intake, glycaemia, insulin sensitivity, lipid flux and inflammatory pathways, several of which may influence both subsequent weight change and liver outcomes. A simple multivariable model that adjusts for treatment‐affected variables could itself introduce bias. Mediation analyses should also account for missing data, as differential missingness in the mediator or hepatic outcome between treatment groups may bias estimates of direct and indirect effects. Missing‐data assumptions should therefore be prespecified and evaluated using appropriate methods and sensitivity analyses. Analyses should ultimately be guided by a prespecified causal diagram and an explicitly defined estimand.

Third, post‐randomization weight‐loss subgroup analyses can remain clinically descriptive and useful, but should not be presented as proof of causal independence. If the scientific question specifically concerns people who would experience limited weight reduction irrespective of treatment assignment, principal‐stratum estimands are conceptually closer to this question than stratification according to observed weight loss. However, principal‐stratum estimands are rarely feasible in typical Phase 2 trials because they require strong assumptions, specialized methods and substantially larger sample sizes. The DD01 study, with only 33 participants assigned to active treatment, illustrates this limitation. Finally, causal and biological evidence should be triangulated. A persuasive claim of weight‐independent hepatic efficacy would ideally combine randomized treatment effects, formal mediation analyses, consistency across prespecified hepatic endpoints and time points and mechanistic or biomarker evidence supporting pathways beyond weight reduction. Useful correlative markers may include serial body weight, appetite or caloric‐intake measures, HbA1c, fasting glucose and insulin, serum lipids, adipokines, inflammatory markers, ALT and AST, ELF score, PRO‐C3, MRE and MRI‐PDFF. Discordance across outcomes might itself be informative. For example, a therapy whose effect on hepatic steatosis is predominantly weight mediated but whose effect on fibrosis is not could reveal clinically important biology that would be obscured by applying a single class‐wide label.

The therapeutic success of incretin‐based medicines makes this distinction increasingly important. Demonstrating hepatic improvement among participants who lose < 5% of body weight is clinically encouraging, but it is not equivalent to demonstrating independence from weight loss. The field should move from threshold‐based subgroup claims towards explicit, endpoint‐specific causal decomposition. Weight‐loss independence is not a phenotype to be observed after randomization; it is a causal question to be estimated.

## Author Contributions

M.Z. drafted the initial manuscript. H.L. and Y.H. contributed to the conceptual development of the figure and critically revised the manuscript. L.C. conceived the Comment, substantially revised the manuscript and supervised the work. All authors reviewed and approved the final manuscript.

## Funding

The authors have nothing to report.

## Conflicts of Interest

The authors declare no conflicts of interest.

## Data Availability

All data are public and can be obtained from the original publications.
